# Control of Appetite and Food Preference by NMDA Receptor and Its Co-Agonist d-Serine

**DOI:** 10.3390/ijms17071081

**Published:** 2016-07-07

**Authors:** Tsutomu Sasaki, Sho Matsui, Tadahiro Kitamura

**Affiliations:** Laboratory for Metabolic Signaling, Institute for Molecular and Cellular Regulation, Gunma University, Maebashi 371-8512, Japan; s.s.s.m.m.m.615@gmail.com (S.M.); kitamura@gunma-u.ac.jp (T.K.)

**Keywords:** anorexia, appetite, d-serine, food preference, hyperphagia, NMDA receptor

## Abstract

Obesity causes a significant negative impact on health of human beings world-wide. The main reason for weight gain, which eventually leads to obesity, is excessive ingestion of energy above the body’s homeostatic needs. Therefore, the elucidation of detailed mechanisms for appetite control is necessary to prevent and treat obesity. *N*-methyl-d-aspartate (NMDA) receptor is a post-synaptic glutamate receptor and is important for excitatory neurotransmission. It is expressed throughout the nervous system, and is important for long-term potentiation. It requires both ligand (glutamate) and co-agonist (d-serine or glycine) for efficient opening of the channel to allow calcium influx. d-serine is contained in fermented foods and marine invertebrates, and brain d-serine level is maintained by synthesis in vivo and supply from food and gut microbiota. Although the NMDA receptor has been reported to take part in the central regulation of appetite, the role of d-serine had not been addressed. We recently reported that exogenous d-serine administration can suppress appetite and alter food preference. In this review, we will discuss how NMDA receptor and its co-agonist d-seine participate in the control of appetite and food preference, and elaborate on how this system could possibly be manipulated to suppress obesity.

## 1. The Importance of Appetite and Food Preference in Obesity

Obesity has become a major health issue on a global scale. Based on the WHO’s definition of obesity (BMI > 30 kg/m^2^), more than 600 million adults were obese and more than 1.9 billion adults were overweight (BMI 25–30 kg/m^2^) in 2014 [1]. Dietary risk factors and physical inactivity, which are two risk factors contributing to the development of obesity, collectively accounted for 10% of global death and disability-adjusted life years (DALYs; sum of years lived with disability and years of life lost) in 2010 [2]. In the Global Burden of Disease Study 2013, 13 among 25 leading global risk factors for DALYs were related to either diet or symptoms of metabolic syndromes, and high BMI itself ranked as the third [3]. Although these global indices draw enough attention, the real impact of obesity is still underestimated, because the obesity risk on disease susceptibility is different among ethnic backgrounds. For instance, the risk of Asians developing diabetes at a BMI of 25 is equal to the risk of Caucasians developing diabetes at a BMI of 30 [4,5]. Therefore, the true number of people at health risk due to obesity around the world could be as high as 2.5 billion (BMI > 25 kg/m^2^), not 600 million (BMI > 30 kg/m^2^) [6].

Obesity is caused by excessive energy intake over energy expenditure [7]. Experimental obesity in man has indicated that humans can control their appetite to achieve the proper weight [8]. However, many of us know from our own experience that humans are prone to overeating, and tend to prefer food that contains high calories. Especially in a developed society where food is easily accessible, food preferences are the first factor influencing the choice of food to ingest [9]. Therefore, to tackle the obesity epidemic, we need to understand the mechanisms controlling appetite and food preference.

## 2. How Are Appetite and Food Preference Controlled?

**Figure 1:**
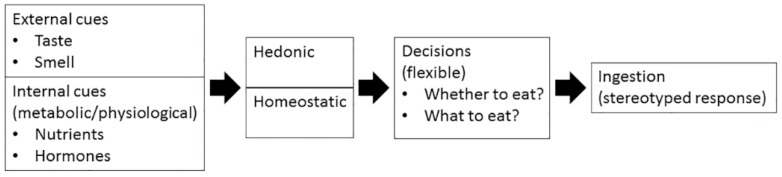
Decision making and feeding behavior.

The motivated behavior, such as feeding behavior, occurs in two phases; the appetitive phase brings animals into contact with food, and the consummatory phase results in ingestion (e.g., chewing, swallowing) [10]. The appetitive behaviors are flexible and non-stereotyped responses, whereas the consummatory behaviors are the final reflexive and stereotyped responses after decisions and efforts have been made to reach the goal object. Therefore, it is important to elucidate how the decisions are made in the appetitive phase, whether to eat and what to eat. Animals make decisions based on the integration of peripheral sensory stimulus (such as taste and smell), internal metabolic and physiological signals that reflecting body’s needs (such as hormones and nutrients), motivation, and experience [11] (Figure 1). There are two major categories for appetite control, homeostatic and hedonic.

**Figure 2:**
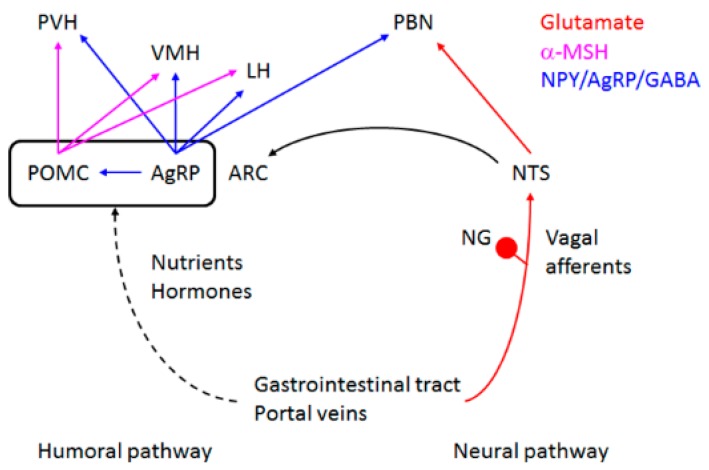
Circuits for homeostatic appetite control. Internal cues are conveyed by humoral and neural pathways and ultimately to the arcuate nucleus (ARC), where the information is integrated. ARC pro-opiomelanocortin (POMC) and agouti-related peptide (AgRP) neurons send projections to the secondary centers. Color of the projections depicts neurotransmitters used. LH, lateral hypothalamus; NG, nodose ganglion; NTS, solitary tract nucleus; PBN, parabrachial nucleus; PVH, paraventricular nucleus of the hypothalamus; VMH, ventromedial nucleus of the hypothalamus.

The homeostatic control of appetite reflects the body’s need for calorie and/or nutrients so that an animal can maintain its body shape. The center for homeostatic control of appetite is located in the hypothalamus, which integrates energy information conveyed from the periphery by nutrients and hormones through two pathways (neural and humoral) [6] (Figure 2). The neural pathway senses peripheral energy status via vagal afferents innervating the gastrointestinal tracts and hepatic portal veins. The vagal afferents relay the nutritional information to the solitary tract of the brainstem and subsequently into the hypothalamus. The humoral pathway is based on the direct input of nutrients and hormones to the arcuate nucleus of the hypothalamus (ARC), which is the primary center for the homeostatic control of appetite. ARC can sense the humoral cues because it is located close to the median eminence, which is permissible to humoral factors for gaining access to the central nervous system. Within the ARC, there are two major types of neurons: anorexigenic pro-opiomelanocortin (POMC) neurons and orexigenic agouti-related peptide (AgRP) neurons. Both types of neurons provide similar projections to the secondary centers for satiety (paraventricular nucleus and ventromedial nucleus of the hypothalamus), and for hunger (lateral hypothalamus). Activity of the target neurons are influenced through the melanocortin type 4 receptors by α-MSH (agonist and the product of POMC through processing) and AgRP (inverse agonist); thus, the balance of activities between POMC neurons and AgRP neurons dictates the activity of secondary centers. AgRP neurons also send inhibitory GABAergic projections to ARC POMC neurons and suppress their activities.

**Figure 3:**
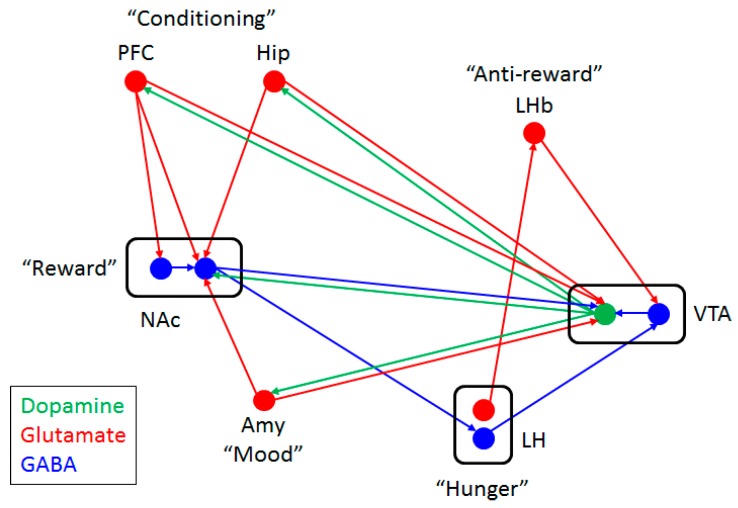
Neuronal circuit for Reward. Functions represented by each node are depicted in quotation marks. Circles indicate soma of neurons with arrows indicating projections. The color depicts which types of neurotransmitters are used. Abbreviations. Amy, amygdala; Hip, hippocampus; LH, lateral hypothalamus; LHb, lateral habenula; NAc, nucleus accumbens; PFC, prefrontal cortex; VTA, ventral tegmental area.

The hedonic component of appetite is comprised of several components, such as enjoyment (core reactions to hedonic impact), wanting (motivation process of incentive salience), and learning (Pavlovian or instrumental associations and cognitive representations) [12]. Food and food-related cues can activate different brain circuits involved in reward processing, including the nucleus accumbens, hippocampus, amygdala, prefrontal cortex, and midbrain. In particular, the mesolimbic dopamine system (dopamine neurons in the ventral tegmental area (VTA) and its mesolimbic projections) promotes the learning of associations between natural reward and the environment [13,14] (Figure 3). In humans, the ingestion of palatable food releases dopamine in the striatum in proportion to the ratings of meal pleasantness and activates reward circuitry [15,16]. Enjoying a reward (“hedonic”, pleasure, palatability) is generated by a small set of hedonic hot spots within limbic circuitry, whereas wanting for a reward (motivation, preference, incentive salience) is generated by a large and distributed brain system [17]. The hedonic enjoyment and motivational wanting signals for a sweet reward are distinctly modulated and tracked in the mesocorticolimbic circuits involving the nucleus accumbens and ventral pallidum, and are separately modulated from Pavlovian prediction (learning) [18]. Consumption of palatable food primes food approach behavior by rapidly increasing excitatory synaptic density onto VTA dopamine neurons [19]. The first exposure to rewarding food increases the firing of dopamine neurons in the VTA, leading to increased dopamine release in the nucleus accumbens [20]. However, with repeated exposure, the dopamine neurons fire when exposure to the stimulus that predicts food delivery, but stop firing when receiving the rewarding food itself [21].

Overall, homeostatic orexigenic signals increase the activity of VTA dopaminergic neurons when exposed to food stimuli, whereas anorexigenic signals inhibit firing of dopaminergic neurons and decrease dopamine release [22]. Neurons in VTA and/or nucleus accumbens express receptors for GLP-1 [23], ghrelin [24,25], leptin [26,27], insulin [26], and orexin [28]. Therefore, hormones that influence homeostatic appetite can also affect the hedonic aspect of appetite. Furthermore, the melanocortin system is capable of regulating mesocorticolimbic activity and food seeking behavior [29]. In summary, internal metabolic and physiological signals can affect both aspects of appetite, and the homeostatic system do communicate with the reward system to control the feeding behavior.

Obesity causes an alteration in multiple phases of appetite: sensing, homeostatic, and reward. Obesity is associated with increased resistance to hormonal actions by the target neurons due to resistances developed at multiple levels, such as blood-CSF barrier, access to receptor-expressing neurons due to gliosis, and intracellular signaling resistance [6]. The decreased capacity to sense the body’s needs and ingested nutritional information through hormones results in overconsumption of food, and altered reward processing [30]. Overconsumption of rewarding food can lead to changes in the reward circuitry [31], causing less activation of the circuits by the palatable food in obese subjects [32]. Blunted activation of the dopamine system by consumption of rewarding food could trigger overconsumption to compensate for the blunted response of the reward circuit [33].

## 3. NMDA Receptor and Its Co-Agonists Glycine and d-Serine

Approximately 70% of synaptic transmission in the central nervous system is carried out by the excitatory neurotransmitter glutamate in the mammalian brain [34,35]. The NMDA receptor is one of the ionotropic glutamate receptors, and is implicated in multiple aspects of brain physiology and cognitive functions, such as learning and memory [36]. It is a heteromeric cation channel made of two GluN1 subunits and two GluN2 subunits [37]. For the efficient opening of the ion channel to remove a magnesium block, the NMDA receptor requires membrane depolarization and binding of co-agonists in addition to the binding of the ligand l-glutamate [38]. Co-agonists bind to the GluN1 subunit, whereas the ligand binds to the GluN2 subunits [39,40,41]. There are two endogenous co-agonists: glycine and d-serine. Although glycine can work as a co-agonist for NMDA receptors and promote excitatory neurotransmission, it also has its own cognate glycine receptors that mediate inhibitory neurotransmission [42]. Furthermore, glycine also affects bile acid conjugation and synthesis of collagen, glutathione, heme, creatine, nucleic acids, and uric acid [43]. On the other hand, the only known biological function for d-serine in vivo is co-agonism toward NMDA receptor [44]. It has a stronger co-agonism toward NMDA receptor than glycine both in vitro and in vivo [45,46]. NMDA receptors are located in neuronal cell membranes at synaptic and extrasynaptic locations. Glycine regulates extrasynaptic NMDA receptors, which contributes to neuronal synchronization, whereas d-serine regulates synaptic NMDA receptors, which are responsible for inducing synaptic plasticity (long-term potentiation and long-term depression) [47].

d-serine is abundant in the forebrain, and its concentration within the brain correlates with the density of NMDA receptor [44,48,49,50]. d-serine production and degradation in vivo are regulated by serine racemase (SR), which converts l-serine into its enantiomer d-serine [51,52,53], and d-amino acid oxidase (DAAO) [54], respectively. The concentration of d-serine in the brain of SR knock-out mice is 10% of that of wild-type mice [55,56], indicating that the local production of d-serine by SR plays a major role in maintaining the local concentration of d-serine and that 10% does come from food and gut microbiota. Indeed, drinking d-serine water for 7 days increased d-serine concentration in the brain of serine racemase KO mice [57], indicating that oral administration of d-serine as a food component could affect the brain d-serine level. Prebiotic feeding, which promotes proliferation of gut microbiota, increased the hippocampal d-serine level [58]. However, the effects of oral d-serine ingestion on gut microbiota remain elusive.

So how could d-serine affect appetite and food preference as a co-agonist for NMDA receptors? Before addressing this question, there is an important molecular and circuit cross-talk to be considered: the glutamate-dopamine cross-talk.

## 4. Glutamate-Dopamine Cross-Talk

Dopamine modulates the functioning of the glutamatergic synapse by acting at different levels [59]. Dopamine modulates and integrates glutamatergic synaptic inputs from the prefrontal cortex, the amygdala and the hippocampus [59]. Dopamine can regulated the activity of ionotropic glutamate receptors leading to reduced AMPA receptor-evoked responses and increased NMDA receptor-evoked responses [60,61,62]. Activation of dopamine D1 receptor usually leads to potentiation of NMDA receptor-dependent currents, while activation of dopamine D2 receptor induces a decrease of AMPA receptor-dependent responses. D1 receptors and NMDA receptors co-exist in synapses of striatal medial spiny neurons, the target neuron of VTA dopamine neurons. Postsynaptic dopamine D1 receptor activates the cAMP pathway, and the activation of the cAMP/PKA/DARPP-32 pathway regulates the phosphorylation status of both AMPA receptors and NMDA receptors, meaning that dopamine signaling can directly control the glutamatergic transmission [63,64,65].

Conversely, glutamatergic inputs also regulate dopamine neurons. The extensive glutamatergic afferents to VTA dopamine neurons from regions involved in the processing of reward (nucleus accumbens), conditioning (amygdala, hippocampus, and prefrontal cortex), and salience attribution (orbitofrontal cortex) modulates the activity of dopamine neurons in response to conditioned cues [66]. The glutamatergic inputs to VTA dopaminergic neurons are organized in a specific manner so that inputs from the prefrontal cortex project onto VTA dopaminergic neurons that project back to the prefrontal cortex and not to other brain regions [67]. Dopamine neurons change their firing from irregular single spike firing to high frequency burst activity in response to unpredicted, biologically salient events and cues that predict rewards, and the induction of the behaviorally relevant burst firing of VTA dopaminergic neurons depends largely on glutamatergic inputs [66]. Glutamatergic projections from specific brain regions differentially affect dopaminergic neurons with different electrophysiological properties [68]. Nucleus accumbens (a target of VTA dopamine neurons) also receive the projections from the amygdala, hippocampus, and orbitofrontal cortex and contribute to conditioned responses to food [69,70]. Therefore, an interwoven relationship exists between the glutamatergic and dopaminergic system. So how could d-serine affect appetite and food preference as a co-agonist for NMDA receptors?

## 5. Control of Appetite and Food Preference by NMDA Receptor and Its Co-Agonist d-Serine

NMDA receptor signaling has been shown to regulate appetite [71,72,73,74,75]. NMDA signaling contributes to the suppression of food intake at multiple appetite-suppressing nodes, such as at the solitary tract nucleus, where the vagal afferents convey peripheral information [76,77,78], at the parabrachial nucleus, where it receives glutamatergic projections from the solitary tract nucleus [79,80], at the secondary satiety centers (ventromedial nucleus of the hypothalamus and the paraventricular nucleus of the hypothalamus [75,81]), and at the lateral habenula, a brain region involved in processing aversive stimuli and negative reward prediction outcomes [82]. Conversely, it is also important in the appetite-promoting nodes, such as in the secondary hunger center (lateral hypothalamic area [83,84,85]) and in VTA promoting reward-based feeding by increasing dopamine release [86,87]. Therefore, NMDA receptor signaling participates in multiple aspects of appetite control: the sensing of peripheral information, homeostatic control, and hedonic control. If so, how does D-serine modulate appetite and food preference?

We tested the effect of ad lib drinking of d-serine water in mice and found that exogenously-provided d-serine suppresses intake of high-preference food [88]. In the absence of food choices, d-serine suppressed the intake of high-fat diet (HFD), high-sucrose diet (HSD), and high-protein diet (HPD), but it did not affect the intake of normal chow at the same concentrations. The effect on suppressing HFD intake was mild at 0.5% (*w*/*v*) but became quite significant at 1.0% so that ad lib drinking of 1.0% and 1.5% d-serine with HFD feeding caused almost a total loss of epididymal white adipose tissue weight. The increasing dose of d-serine was associated with increased incidences of mice found dead due to probable starvation. These data indicate that there is a very narrow therapeutic window for using d-serine safely as an appetite-suppressant. Interestingly, significant suppression of food intake and starvation incidences were similarly observed among HFD, HSD, and HPD, indicating the phenotypes caused by d-serine were not dependent on a particular macronutrient. 

In the presence of two food choices, d-serine altered food preferences in all three food types tested (HFD, HSD, and HPD). d-serine reversed the food preference when it was given at the acquiring phase of food preference (d-serine ingestion was initiated simultaneously with new food choices), but it only canceled the expressed food preference (d-serine ingestion was initiated after mice acquired the preference for food under two choices). Contrary to the starvation effect observed in the absence of food choices, the total caloric intake was not suppressed in the presence of food choices. The preference-reversing effect of d-serine is dependent on co-agonism toward NMDA receptor, because the effect was canceled in the presence of the co-agonist site-specific NMDA receptor inhibitor l-701,324. Furthermore, using the fasting/re-feeding experiment, under which condition mice prefer high-carbohydrate diet (like normal chow diet) over HFD, we showed that d-serine suppressed the intake of normal chow more effectively than HFD at the first hour of re-feeding, but by the third hour d-serine suppressed the intake of HFD only. These data also support the idea that the effect of d-serine is not dependent on a particular macronutrient. Rather, d-serine suppresses the intake of food that mice prefer to eat at a given moment.

So where does d-serine work to suppress the intake of high-preference food? Using capsaicin-treated sensory-deafferented mice, we further showed that the effect of d-serine on reversing preference for HFD was not dependent on sensory and vagal afferents. Therefore, d-serine is likely to work in the central nervous system. The concentration of d-serine within the brain increases by several fold from the endogenous level after 48 h of ad lib drinking of 1.5% d-serine (unpublished data), the timing of which corresponds to the timing of phenotypes observed. d-serine does not require intact leptin receptor signaling for its effect because it is also effective in genetically obese *db*/*db* mice that lack leptin receptor signaling [88]. Collectively, the main target of d-serine in the context of controlling appetite and food preference appear not to be at the level of sensing (both peripheral and metabolic) or homeostatic phase, but to be at the hedonic phase. It is probably not affecting the enjoyment component of a hedonic appetite, because mice that do not prefer HFD under d-serine drinking did not show aversion to HFD. Therefore, it is likely that d-serine is affecting wanting (motivation process of incentive salience), and/or learning (Pavlovian or instrumental associations and cognitive representations). However, the precise target neurons and circuits need to be identified by further investigations.

## 6. Can d-Serine Be Used to Prevent Obesity?

Obesity cause alteration in multiple systems (sensory, homeostatic, and reward), and multi-level approaches are needed to re-balance the systems and achieve normal weight. Glutamate-dopamine cross-talk implies that d-serine may modulate the reward system through the cross-talk, and d-serine was effective in altering parts of the reward aspect of appetite at least in mice [88]. So can d-serine be used to prevent obesity in humans?

d-Serine has been administered peripherally (orally, intraperitoneally, or subcutaneously) as supplementation to schizophrenic patients, who are known to have low brain d-serine levels, with improvements in some of the symptoms [89,90,91,92]. The doses of d-serine used in the clinical trials for schizophrenia are between 30 to 120 mg/kg/day orally [93,94]. The dose used in our murine study would be 1.5 to 7 g/kg/day orally using 1.5% d-serine water [88], at least more than 10-fold higher than the doses used in the clinical trial without any side effect. Can this amount of d-serine be ingested through natural food products? High concentration of d-serine was detected from Japanese black vinegars, fermented milks and yogurts [95]. Passion fruit contains a high amount of d-serine (approximately 17 μmol/L) and so does black ripe olive (4 mg/100 g fresh weight) [96,97]. However, you would have to drink roughly 1 kL/kg body weight per day of passion fruit juice or 10 kg/kg body weight per day of black ripe olive to achieve the amount of d-serine ingestion observed in our experiment. Therefore, it is impractical to obtain d-serine from food only, and it is probably necessary to ingest as a supplement. At least, d-serine tastes sweet to humans [98]. So can it be used safely and effectively used as a dietary supplement to control appetite and food preference in obese subjects?

Because the effect of d-serine on appetite was clear with 1.0%–1.5% but quite mild with 0.5% in rodents, the confirmed safe dose (used in human clinical trials) may not be sufficient to achieve the effect on appetite and prevent obesity. There are concerns for increasing the dose of d-serine administered, because high d-serine levels may be proposed as potential causes for some diseases, such as neuropathic pain [99] and amyotrophic lateral sclerosis [100,101,102,103]. d-serine may also promote depression, which is frequently associated with obesity, because NMDA receptor antagonists are currently considered as an important alternative antidepressant option in major depression [104]. So cautions are required to test the use of d-serine in obesity prevention. Another issue is that we found that mice re-gained the preference for HFD, HSD, and HPD to the pre-treatment levels within five days after stopping d-serine ingestion [88]. So it would require continuous d-serine ingestion to alter the expressed preference for particular food. The potentially narrow therapeutic window, untested long-term consequences, and concerns for possible side effects are the major limitations.

In conclusion, it is premature to start using oral d-serine administration to control feeding and prevent obesity in humans. There are numerous hurdles that need to be cleared before it can be used safely and effectively in preventing obesity in humans. Feeding behavior phenotypes observed in mice by d-serine ingestion is quite phenomenal, and NMDA receptor co-agonist might be therapeutically efficacious. It is necessary to identify the responsible neural circuits and elucidate the exact mechanisms for the d-serine’s effect on feeding behavior. Impaired NMDA receptor signaling assisted by endogenous d-serine in the nucleus accumbens is implicated in cocaine addiction [105,106]. It may be one of the parallel mechanisms between drug addiction and food addiction-like behavior in obesity. Further study can provide a basis in the search for alternative co-agonist molecules that have similar efficacy but with a much better side effect profile.

## Abbreviations

NMDA receptor	*N*-methyl-d-aspartate receptor
WHO	World Health Organization
BMI	body mass index
POMC	proopiomelanocortin
AgRP	agouti-related peptide
α-MSH	alpha melanocyte-stimulating hormone
GABA	gamma amino butyric acid
VTA	ventral tegmental area
GLP-1	glucagon-like peptide-1
CSF	cerebrospinal fluid
SR	serine racemase
DAAO	d-amino acid oxidase
cAMP	3′,5′-cyclic adenosine monophosphate
PKA	protein kinase A
DARPP-32	dopamine- and cAMP-regulated phosphoprotein, molecular weight 32 kilo Dalton
AMPA	α-amino-3-hydroxy-5-methyl-4-isoxazolepropionic acid
HFD	high-fat diet
HSD	high-sucrose diet
HPD	high-protein diet
